# Evaluation of Heavy Metals and Microbiological Contamination of Selected Herbals from Palestine

**DOI:** 10.1515/biol-2019-0050

**Published:** 2019-11-15

**Authors:** Murad Abualhasan, Nidal Jaradat, Zahraa Sawaftah, Hala Mohsen, Dyala Najjar, Wahbi Zareer

**Affiliations:** 1 An-Najah National University, Faculty of medicine and health sciences, Department of Pharmacy, Nablus, Palestine; 2 Quality control manger, Birzeit Palestine pharmaceutical company, Ramallah, Palestine

**Keywords:** herbal medicine, heavy metals contamination, microbiological contamination

## Abstract

**Background:**

Herbal medicine is widely used for the prevention and treatment of diseases worldwide including Palestine and may require long term usage. The level of some heavy metals and microbial contaminants in some of these medicinal plants consumed by Palestinians were studied in order to evaluate their quality.

**Methodology:**

The level of metals including: Zinc, Cadmium, Lead and Copper were quantified by Atomic absorption spectroscopy (AAS). Moreover, the bacterial and fungal contaminations were tested for some of the selected plants in Palestine. The procedures of microbial and elemental testing of the plants followed USP.

**Results:**

The result of the heavy metals testing showed that copper and cadmium were above the allowable limits in all the tested plants. Zinc metal was above the allowable limit in 78.9% of the tested samples. The microbiological results of the tested plants showed that 63.2% of the tested plants were contaminated by bacteria and 89.5% were contaminated by yeast.

**Conclusions:**

Herbal medicine used in the Palestinian markets doesn’t meet the international requirement for heavy metal and microbiological limits. Therefore, urgent action has to be taken by the responsible authorities including the Ministry of health to implement importation and registration requirements and perform regular quality checks of sold and imported herbal medicines. Pharmacists as expert professionals must take an active role in selling and advising consumers about the quality and efficacy of the sold plants.

## 1 Introduction

Large sections of the population in developing countries still rely on herbal medicines for their primary care. Traditional medicine is widely used for the prevention and treatment of many diseases, and are also used to boost energy and improve the immune system [1, 2].

In many countries including Africa, India and China their populations rely on traditional medicine to meet their health care needs [3]. Nowadays herbal medicines are widely present in herbal traditional shops, supermarkets as well as community pharmacies. The herbal medicine is present in different forms such as herbal bags, creams, powder etc. In Palestine, herbal medicine is considered an integral part of Palestinian culture and plays an important role in current public healthcare. The Palestinian hills and mountains are covered with more than 2600 plant species of which about 700 of them are of known medical uses [4].

The limits of toxic metals in the form of impurities depend on the nature of the sample and the contaminants or residues. The national limits for toxic metals and microbial contamination in various types of herbal products are different for each country and depend on the herb type and whether it is raw material or a finished product [5].

The WHO has put specification for microbial limits including aerobic bacteria, yeast and Escherichia coli. The limit varies according to the intended use of the herbs. In general the allowable limits are more when the herbs are intended for external use or require boiling before administration [6].

The use of medicinal herbs in therapeutic remedies is a common practice among Palestinians. Many of the herbs used are mainly cultivated and produced in Palestine; these herbs include: Sicilian Sumac, Oleander, Palestinian Arum, Hawthorn and Cardamom [7]. However, many of the herbs used are imported from African countries such as Egypt or from South Asia such India. Herbal medicine in Palestine is widely used in the treatment of GI Disturbances, cardiovascular, sedation and weight control [8, 9, 10].

Some of the widely used herbs in traditional medicine in Palestine include: *Matricaria chamomilla* L. (Chamomile) is a plant with white flowers, it is commonly used for the relief of inflammation, ulcers, muscle spasm, gastrointestinal disorders, hemorrhoids, menstrual disorders and insomnia [11]. *Pimpinella anisum* L. (Anise) has small green to yellow seeds, it is commonly used as muscle relaxant, analgesic and has beneficial effects for dysmenorrhea and menopausal hot flushes in women. In diabetic patients, aniseeds have a hypoglycemic and hypolipidemic effect [12]. *Zingiber officinale* Roscoe (Ginger) is the horizontal stem from which the roots grow and the main portion of it consumed. Ginger is commonly used for arthritis, rheumatism, muscular aches, sore throats, cramps, constipation, indigestion, vomiting, hypertension, dementia and have anti hyperglycemic effect [13]. *Crataegus azarolus* L. (Hawthorn) is a low, dense, spiny tree with orange fruit the fruits, flowers and leaves are the parts used. It is used for control of hyperglycemia with its associated complications[14]. *Hibiscus sabdariffa* L. (Roselle) is fleshy and develops bright red flowers as the fruit matures. It has antioxidant, hypotensive, and anti-atherosclerotic effects [15].

Herbs in Palestine are usually sold in the traditional medicine shops and some of them are sold in pharmacies. The Ministry of Health (MoH) is the authority that monitors the medicinal plants market in Palestine. The rules to control the herbal medicines are newly established. The general directorate of pharmacy in MoH is the department that takes the responsibility of importing, exporting and producing the medicinal herbs in the Palestinian market. A set of rules has been recently issued which include that any store selling or importing herbs must be licensed by the ministry of health. The store must employ a responsible pharmacist, chemist, medical biologist, food engineering or chemical engineer. The ministry of health has the right to withdraw any herb proved to have a dangerous side effect based on WHO recommendations. However, the MoH have not yet established rules and limitations for bacterial and elemental contamination of herbs. The wide used of herbal medicine in Palestine makes it necessary to perform regular investigation and to make a quality control check for the microbial and heavy metal content of herbs sold in the Palestinian markets [16].

Many studies were conducted all over the world to evaluate herbal heavy metal and microbial contamination. Studies were conducted in countries like Ghana, Japan, Ayurveda, Nigeria, Brazil, North Botswana, South Africa and some of the Arabian countries such as Saudi Arabia and Egypt. The main objective of these studies was to analyze metals such as lead (Pb), cadmium (Cd), Manganese (Mn), mercury (Hg), and arsenic (As), in some of the most commonly used medicinal plants. The results of these studies showed that metals like Lead , Cadmium, Aluminum, Mercury and arsenic in the tested plants were above the set standard [17, 18, 19, 20].

In this study we performed a quality check of the microbial and heavy metal contamination of some selected herbs that are widely used among the Palestinian population. The study will reflect the actual situation of the herbal medicine in Palestine and will raise the herbal contamination issue with the concerned authorities. The results of this study will come up with recommendations and advice to the concerned authorities to take the necessary corrective actions.

## 2 Material and Methods

### 2.1 Chemical and Reagents

All the reagents used were purchased from reliable resources, the following reagents were used throughout the research project: Nitric Acid 70% (Riedel-de-Haen™; Germany), Perchloric Acid 70% – 72% (Riedel-de-Haen™; Germany) were used in acid digestion of herbals, Tryptic Soy Agar ( Difco ™; France), Sabouraud Dextrose Agar (Difco™; France) were used in media preparation, Tryptic Soy Broth (Difco™; France), Tween80 (Difco™; France) were used in Fluid 3 preparation used in the microbiological testing.

### 2.2 Instrumentations

Hotplate (Model LMS-1003; Lab Tech) was used in acid digesting of the collected herbs. An Atomic Absorption Spectrometer (Model ICE3000; Thermo Scientific) was used in the quantitative analysis testing of heavy metals. Autoclave (Model DLOV 3764; DE-Lama) was used in sterilization of fluid 3 (F3) and Ager. Laminar Air Flow (BBS-V1300; Biobase), Incubators (BC3100-R1; Biorold) were used in microbiological testing of herbal samples, and a Vortex (Genie 2 SI) was used for sample mixing.

### 2.3 Plant Collection

Five medicinal plants that are widely used in Palestine were collected from popular herbal selling stores and from community pharmacies which sell traditional herbal medicine, these plants include: *H. sabdariffa* (Roselle), *P. anisum* (Anise), *M. chamomilla* (Chamomile), *C. azorolus* (Hawthorn), and *Zingiber officinale* (Ginger). Botanical characterization was conducted at An-Najah National University in the Pharmacognosy Laboratory and kept under the herbarium voucher specimen numbers: Pharm-PCT-1195, Pharm-PCT-2768, Pharm-PCT-178, Pharm-PCT-712, and Pharm-PCT-2724.

The sources of the selected plants were from different places, for example: Hawthorn is a Palestinian native plant, so all of the collected samples were harvested from Palestine. Anise was imported from south Asian countries, Roselle and Chamomile were imported from Egypt.

All the samples were grinded to fine particles to a powder form in our research lab. They were put it in a glass container and were stored in a dry, dark and cool place during the project period. The total numbers of the herbal samples collected and analyzed were nineteen samples.

### 2.4 Elemental analysis

#### 2.4.1 Acid Digestion of the herbal sample

Acid Digest was prepared by oxidizing 0.2 g of the dry powder plant with 10 ml of an acid mixture of (2:1) Nitric acid: Perchloric acid and the powdered plants were stirred with the mixture for overnight. The mixture was then filtered; 1 ml of the filtrate was diluted to 20 ml of distilled water. The diluted samples were used in the quantitative measurement of Lead (Pb), Cadmium (Cd), Cupper (Cu) and Zinc (Zn) by using Atomic Absorption Spectrophotometer. The analysis was performed in triplicate for every herbal sample [21].

#### 2.4.2 Elemental quantitative analysis

Three point standard concentrations were prepared for each metal to be analyzed. The samples were then diluted with water to be within the calibration curve range. The concentrations of the samples were determined using the calibration curve regression equation [22]. The three-point calibration concentration for each measured metal is illustrated in **Table 1**

**Table 1:** Calibration concentrations of measured metals

	Metal (ppm)			
	**Zinc ( Zn )**	**Cadmium (Cd)**	**Lead (Pb)**	**Cupper (Cu)**
Standard #1	0.100	0.050	0.250	0.100
Standard #2	0.300	0.100	0.500	0.300
Standard #3	0.500	1.000	1.000	0.500

### 2.5 Microbiological testing

Bacterial and fungal contamination were tested using procedures described in the United State Pharmacopoeia-USP [23].

#### 2.5.1 Media Preparations

Fluid 3 *“F3” preparation*: Fluid 3 was prepared by adding 15 g Tryptic Soy Broth in 500 ml distilled water then 1 ml of Tween 80 was added. Water was added gradually to a final volume of 1000 ml,

*Tryptic Soy Ager Preparations*: This media was prepared by suspending 40 g of the powder in 1 L of Purified water. The mixture was thoroughly stirred with heat and was boiled for one minute to dissolve the powder completely. This media was used for bacterial growth.

*Sabouraud Dextrose Ager Preparation*: This media was prepared by dissolving 65g of the powder in 1 L of purified water, mixed thoroughly with heat and frequent agitation to dissolve the powder completely. The agar media was autoclaved by heating it at 121°C for 15 minutes. This media was used for cultivation of fungi. The media was put it in the oven at 50°C to keep it liquid and to use it when needed.

#### 2.5.2 Sample transplantation and incubation

A dry herbal sample (3 g) was added to Fluid 3 (30 g). Serial dilutions of the samples were prepared: (10^-1^), (10^-2^), (10^-3^) and (10^-4^). Each dilution was inoculated at Tryptic Soy Ager plates which were incubated at (30–35°C) for 48 hours for bacterial identification. Fungal microbial testing was performed by inoculating the prepared dilution on Sabouraud Dextrose Ager plates which were incubated at (20-25°C) for 5 days. The results were reported as counts of colony forming (cfu) per each gram or ml [23].

### 2.6 Statistical analysis

Statistical Package for the Social Sciences (SPSS) software program was used to perform descriptive analysis. Analysis of variance (ANOVA) test was used to test if there was a statistical significant different between means. Pearson’s chi-squared test was used to determine whether there is a significant difference between the expected frequencies and the observed frequencies in more than one categories. In all the mentioned statistical test if the p-value is less than 0.05, the null hypothesis was rejected and it was concluded that a significant difference does exist.

## 3 Result and discussion

### 3.1 Descriptive analysis

The number of collected samples of each herbal type was almost equal (Average= 4) and there was no variability in the size of collected plants. The plants were collected from five different selling stores. The number of collected samples from the selling was equally distributed. Hawthorn was originally brought from Palestine, Anise was imported from India. Chamomile, Roselle and Ginger were imported from Egypt.

### 3.2 Results of heavy metals

The results for heavy metals of the tested plant are illustrated in **Table 2** The results showed that elements like copper and cadmium were above the allowable limits in all the tested plants; the average value was 56.52 and 64.01 ppm respectively. In contrast, lead was within the acceptable limits in all the tested plants (average =0.3210 ppm).

**Table 2:** The result of heavy metals

	Result	Frequency (%)	Average
Metal (limit)			(ppm)
**Cu**	Fail	19 (100.0)	56.52
**(20 ppm)**	Pass	0 (0)	
**Cd**	Fail	19 (100.0)	64.01
**(0.3 ppm)**	Pass	0 (0)	
**Zn**	Fail	15 (78.9)	162.76
**(50 ppm)**	Pass	4 (21.1)	
**Pb**	Fail	0 (0)	0.3210
**(10 ppm)**	Pass	19 (100.0)	

The results of zinc levels were compared in different plants types and were statistically tested if there was a significant difference between different types of plants **(Table 3)**. The results revealed that there was a difference in zinc level between herbal plants (p=0.033). However, the zinc level results showed that there was no significant difference between selling stores (p>0.05). This result showed that all the selling stores in the Palestinian market were selling a similarly low quality of herbs. This may be attributed to the improper storage conditions in all herbal shops and could be due to importing a low quality of herbs from international suppliers. The concerned authorities have to take an active role to approve and license for an imported herbal medicine before it reaches the final customer.

**Table 3:** Zinc result in different plants

	Plant					Total
	Hawthorn	Chamomile	Roselle	Anise	Ginger	
Pass	1	0	0	3	0	**4**
Fail	2	4	4	1	4	**15**
Total	3	4	4	4	4	**19**

Contamination of herbs by toxic metals can be attributed to many causes including: environmental pollution, soil composition and using fertilizers [24, 25]. Some of the metals such as Cu, Cd and Zn had a higher concentration than the allowable level, this can be attributed to the use of fertilizers & pesticides which contain these elements in their compositions [26, 27]. By contrast, for the last few years manufacturers of fertilizers have not used Pb; thus, the concentration of Pb in the soil dropped sharply in the last years [28]. Moreover, the reduced use of pewter and the gradual decrease of lead solder in cans and the replacement of lead water pipes carrying water in cities and towns has led to less lead contamination in soil & water [29, 30].

### 3.3 Microbiological testing

**Figure 1:**
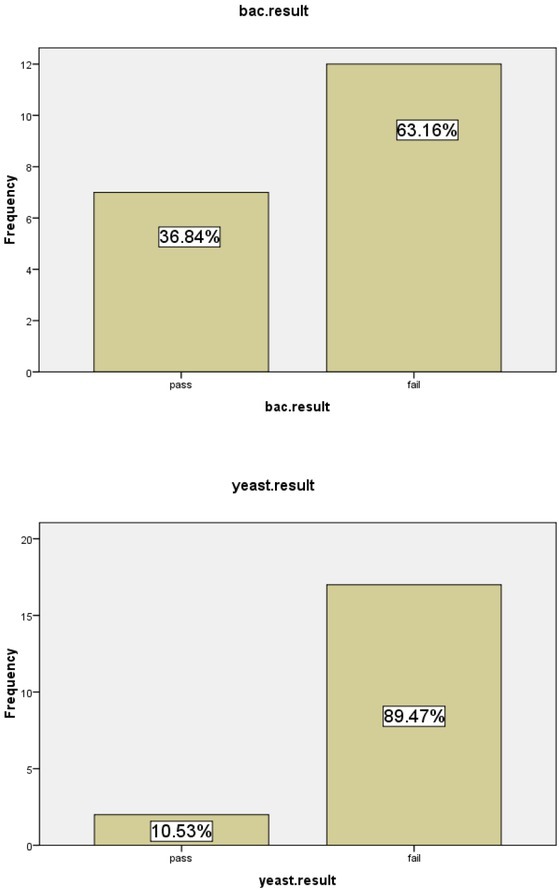
The microbiological result of the tested

The microbiological result showed that the bacterial count was over the limit in 63.2 percent of the tested plants and yeast was over the allowable limit in 89.5 percent of the total tested plants. The colony counts in the majority of the failed herbals were too many to count (TMC). The detailed microbiological results are illustrated in (**Figure 1)**

**Figure 2:**
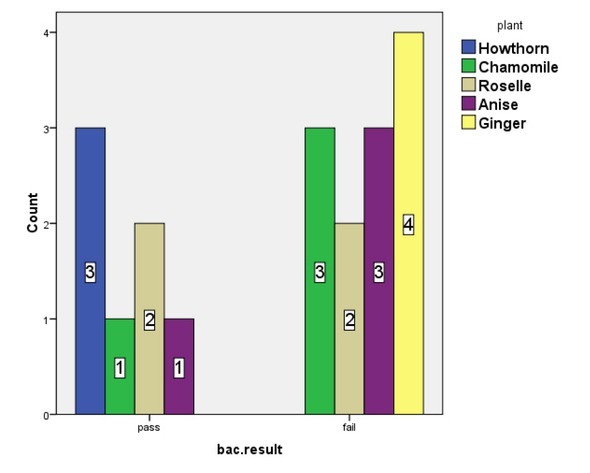
Bacterial Chi Square results

We further investigated if the bacterial results were significantly different for the tested plants. The Chi square statistical test was performed and the bacterial results revealed that there was no significant difference between the plants (p=0.83). However, the bacterial contamination was significantly different among the selling stores (p=0.03). The above results demonstrate that the variation in bacterial results among the selling stores could be due to their difference in handling and storage practices of the herbs they sold (**Figure 2)**.

## 4 Conclusion

Herbal medicines used in the Palestinian markets don’t meet international requirements. Urgent action has to be taken by the responsible authorities such as implementing importation and registration requirements. The sellers of the herbal plants must undergo regular quality checks. The results revealed a variation among the herbal sellers especially in microbiological contamination which may be due to a difference in handling and storage practice. An awareness program for both sellers and consumers about the danger of using contaminated herbs must be implemented. Pharmacists and community pharmacies must take an important role in selling and advising the consumers about quality, storage and medical effects of the sold plants.
